# Editorial: Synthetic Biology-Guided Metabolic Engineering

**DOI:** 10.3389/fbioe.2020.00221

**Published:** 2020-03-20

**Authors:** Rodrigo Ledesma-Amaro, Pablo I. Nikel, Francesca Ceroni

**Affiliations:** ^1^Department of Bioengineering, Imperial College London, London, United Kingdom; ^2^Imperial College Centre for Synthetic Biology, Imperial College London, London, United Kingdom; ^3^The Novo Nordisk Foundation Center for Biosustainability, Technical University of Denmark, Lyngby, Denmark; ^4^Department of Chemical Engineering, Imperial College London, London, United Kingdom

**Keywords:** synthetic biology, metabolic engineering, genetic constructs, bioproduction, TX-TL

Synthetic biology can be now considered a mature discipline. A growing number of diverse synthetic gene constructs and circuits have been designed within a wide number of organisms leading to a profound impact on different fields. This Research Topic features and reviews some of the latest progress in Synthetic Biology applications and improvements within the Metabolic Engineering portfolio, covering different aspects in the domain (e.g., bioinformatics, design of synthetic pathways, and implementation of multi-omics approaches).

Among the successes of Synthetic Biology and Metabolic Engineering, the ability to achieve smarter construct design and higher yields of valuable chemicals needs to be considered. As one example, Callari et al. engineered *Saccharomyces cerevisiae* to produce the diterpene casbene, precursor of many terpenoids of medical interest. The authors successfully achieve increased casbene titers via expression of heterologous enzymes that can boost internalization and conversion of precursors; performing dynamic control of inducible promoters also helped maximize the pathway flux toward casbene production. Another example comes from 3-hydroxypropanoic acid (3-HP), a valuable product employed in the bioproduction of several other chemicals, including bioplastics. Maury et al. achieved high product titers in batch cultivations using engineered *S. cerevisiae*. Firstly, the authors characterized a series of 34 native promoters which respond to glucose. Placing the 3-HP pathway under the control of a promoter active in absence of glucose, they then achieved decoupling of biomass and compound production, maximizing product formation. Jers et al. reviewed the state-of-the-art in 3-HP production *via* Metabolic Engineering. The review highlights how major improvements have been reached but that efforts are still needed to achieve a scalable system, mainly relying on better biochemical characterization of the synthesis pathways and bioprocessing conditions. In addition, Frost et al. present an important case of cooperation between Synthetic Biology and Metabolic Engineering approaches in the recombinant production of hemoglobin. Authors summarize the state-of-the-art and the challenges that this technology is facing while suggesting novel routes to improve the promising yeast-based production of artificial blood.

Despite the great successes, there is still a wide number of organisms that could become valuable hosts for the production of important chemicals but that still suffer from the lack of properly standardized bio-parts for their easier engineering. A valid example on this respect comes from Rajkumar et al.. The authors developed a toolkit of standardized genetic parts, namely promoters and terminators, for the genetic engineering of a non-conventional yeast with high potential, *Kluyveromyces marxianus*. Parts were isolated from the genome, “domesticated” for golden gate assembly and then cloned for characterization under different conditions. Another example is presented by Santo-Merino et al. where the authors summarize the latest efforts in the development of novel biological parts for engineering cyanobacteria.

One of the reasons why we still miss parts and tools for a more rapid engineering of many organisms is our limited knowledge on many of them. Together with collecting new information, we need progress on how to handle and use it. García-Granados et al. suggest that in the era of the “—omics” approaches we are in, mathematical modeling and biocomputing could serve as essential tools to coordinate and predict information, providing deeper and more accessible understanding that we could use for engineering target hosts. In this respect, Goñi-Moreno and Nikel advocate the *in silico* conceptualization of metabolism and its motifs as the way forward to achieve *whole-cell biocomputations*. The authors argue that the design of merged transcriptional and metabolic circuits will not only increase the amount and type of information being processed by a given synthetic construct, but that it will also provide fundamental control mechanisms for increased reliability on synthetic implants.

Not only handling information is challenging, but also making sure that this information is accurate and the data we collect are robust. Jessop-Fabre and Sonnenschein give an overview of the efforts currently in place toward a more reproducible science. Naming novel developed bio-foundries and cloud-laboratories together with new effort for the standardization of protocols and results, the authors suggest the way Synthetic Biology should continue to invest in to achieve more reliable data acquisition. One example comes from Nadler et al.. Here, the authors develop a novel system, named CopySwitch, that allows rapid transformation and copy number modulation of any genetic construct in an easier way than previously possible in *Bacillus subtilis*.

Again, López et al. considered two different *S*. *cerevisiae* strains engineered for β-carotene production comparing their growth in shake-flasks and in bench-scale fed-batch fermentation (as a proxy for industrial bioprocessing conditions). Surprisingly, the two strains showed opposite behavior, highlighting the need for proper understanding of how engineered systems behave in the final, industrially relevant settings.

An important source of information and characterization is represented by transcription and translation systems (TX-TL). In their contribution, Koch et al. describe how TX-TLs offer powerful tools for the prototyping of genetic constructs and understanding of network behavior out of the cellular context. This could be of great importance in Metabolic Engineering for the modeling and prediction of the behavior of entire pathways so to optimize them prior *in vivo* engineering. However, preparation of TX-TL extracts is still challenging, and automation tools could help improve reproducibility. Combination of TX-TL technology and liposome technology has recently gained momentum, and many successful examples in this emerging field are based on genetic constructs that can be expressed in a “simplified cell” able to interact with living cells and communicate with them. On this premise, Rampioni et al. present the latest advances in synthetic cells development. The possible applications of such a technology are many, as many are the opportunities to use it for better understanding biological systems.

In all, the works collected in this Research Topic expose that we are experiencing critical times for Synthetic Biology-guided Metabolic Engineering, where deeper and better understanding of the complexity of biological systems enables more predictable designs. Walking this pathway will significantly improve the applicability of first-design principles for living organisms toward reliable cell factory design, construction and testing.

## Author Contributions

All authors listed have made a substantial, direct and intellectual contribution to the work, and approved it for publication.

### Conflict of Interest

The authors declare that the research was conducted in the absence of any commercial or financial relationships that could be construed as a potential conflict of interest.

